# An update on source-to-sink carbon partitioning in tomato

**DOI:** 10.3389/fpls.2014.00516

**Published:** 2014-10-06

**Authors:** Sonia Osorio, Yong-Ling Ruan, Alisdair R. Fernie

**Affiliations:** ^1^Instituto de Hortofruticultura Subtropical y Mediterránea “La Mayora”, Department of Molecular Biology and Biochemistry, University of Malaga – Consejo Superior de InvestigacionesCientíficas, Málaga, Spain; ^2^Max-Planck-Institut für Molekulare PflanzenphysiologiePotsdam-Golm, Germany; ^3^Australia–China Research Centre for Crop Improvement, The University of NewcastleCallaghan, NSW, Australia; ^4^School of Environmental and Life Sciences, The University of NewcastleCallaghan, NSW, Australia

**Keywords:** tomato, carbon partitioning, source organs, sink organs, carbohydrates

## Abstract

Plant growth and carbon metabolism are closely associated since carbohydrate in the form of sucrose generated by photosynthesis, provides the primary source of building blocks and energy for the production and maintenance of biomass. Regulation of carbon partitioning between source and sink tissues is important because it has a vast influence on both plant growth and development. The regulation of carbon partitioning at the whole plant level is directly linked to the cellular pathways of assimilate transport and the metabolism and allocation of sugars, mainly sucrose and hexoses in source leaves, and sink organs such as roots and fruit. By using tomato plant as a model, this review documents and discusses our current understanding of source–sink interactions from molecular to physiological perspectives focusing on those that regulate the growth and development of both vegetative and reproductive organs. It furthermore discusses the impact that environmental conditions play in maintenance of this balance in an attempt to address the link between physiological and ecological aspects of growth.

## INTRODUCTION

The partitioning and allocation of carbon (C) is intimately connected to plant growth since the export of carbohydrate from photosynthesizing leaves provides the substrate for the growth and maintenance of non-photosynthetic tissues. Through photosynthesis plants can highly efficiently convert CO_2_ into 3-phosphoglyceric acid and glyceraldehyde-3-phosphate leading to the biosynthesis of sugars as well as terpenoids and fatty acids. This fixed carbon is transformed into reserve molecules, which can be broken down at a later time to provide the cell with ATP, reducing power, and carbon skeletons, which support a number of physiological functions including growth. Carbohydrates such as sucrose provide both an energy source and the building blocks for the production and maintenance of biomass.

Biomass accumulation in plants is a remarkably stable function of light intercepted by the canopy and CO_2_ transformation into dry matter via photosynthesis, thus illustrating the dependence of plant growth on C fixation. Photosynthetically active “source” tissues such as mature leaves, export fixed C, primarily in the form of sucrose, to non-photosynthetic “sink” tissues such as fruits or reproductive organs, tubers, meristems, or roots (Koch, 2004). During its life cycle, a typical plant undergoes considerable changes in the dynamics of carbon transport and metabolism in both source and sink organs as well as in the degree of competition among various sinks for the common pool of carbohydrates available. Changes in source and sink activities are known to induce cyclic patterns of growth (production flushes; Gary et al., 1993; Valentin et al., 1998; Gautier et al., 2001; Bertin et al., 2003). Commercial horticultural crops such as citrus and apple are exposed to a sustained pruning throughout their growth cycles in order to control their growth and to maintain a desired balance of photoassimilate partitioning between source and sink organs. However, altered source sink dynamics across development are by no means confined to crop species with several reports evidencing this behavior in non-cultivated species (Graf et al., 2010; Pyl et al., 2012; Sulpice et al., 2014).

**FIGURE 1:**
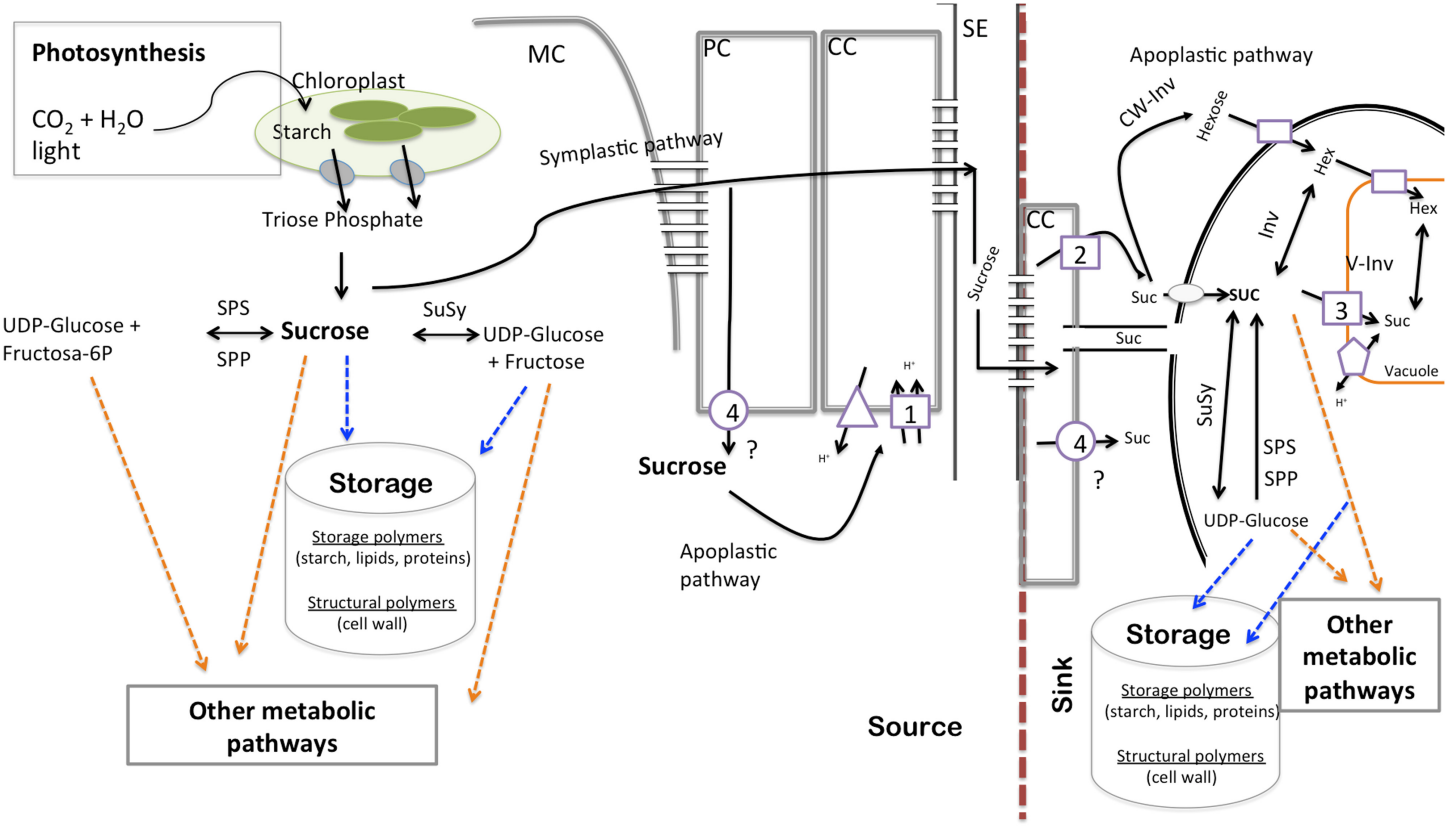
**Schematic diagram of transfer and transport processes contributing to the flow of assimilates through the source–sink system.** Circles describe facilitators, squares represent symporters, triangles describe H^+^-ATPases/PPases, and pentagons antiporters. 1, *LeSUT1*; 2, *LeSUT2*; *LeSUT4*; 4, putative *SlSweets.* MC, mesophyll cell; PC, parenchyma cells; CC, companion cells; SE, sieve element; Suc, sucrose; SPS, sucrose-P-synthase; SPP, sucrose-P-phosphatase; CW-Inv, cell wall invertase; V-Inv, vacuolar invertase.

In order to fully understand the relationship between photoassimilate partitioning and growth, we need to consider three important key steps, (1) production of photoassimilates (source capacity), (2) transport of photoassimilates, and (3) utilization of photoassimilates in sink organs (**Figure 1**). This brief review highlights the role of carbohydrate transport and metabolism in plant growth and its perspective in altering agronomic yield. For this purpose we focus on recent development in tomato, which as well as being an important horticultural crop is a model for research on source–sink interactions and competition.

## CARBOHYDRATE PARTITIONING IN SOURCE TISSUES

The photosynthetic activity of source tissues is determined by the activity of various enzymes of the Calvin–Benson cycle, which can be divided into three distinct phases. In phase 1 (carbon fixation), CO_2_ is condensed with the five-carbon sugar ribulose bisphosphate. This reaction is catalyzed by ribulose 1,5-*bis*phosphate carboxylase (Rubisco) generating two molecules of 3-phosphoglycerate (3-PGA). In phase 2 (reduction), the 3-PGA is converted to glyceraldehyde 3-phosphate, the three-carbon precursor of sucrose. In phase 3 (regeneration), the ribulose bisphosphate is regenerated in order to incorporate CO_2_ to initiate the cycle. In order to remobilize the inorganic phosphate incorporated in the primary products of photosynthesis, assimilates are converted either to sucrose in the cytosol, or to transitory starch, which is synthesized in the plastids and degraded into glucose and maltose at night. Starch can be seen as an overflow product synthetized when the rate of CO_2_ fixation exceeds the rate of sucrose synthesis. Feedback inhibition of sucrose synthesis via the signal metabolite fructose-2,6-bisphosphate leads to the accumulation of phosphorylated intermediates and decrease inorganic phosphate (Pi) in the chloroplast, resulting in activation of ADP-glucose pyrophosphorylase (AGPase) by a rising glycerate-3-phosphate:Pi ratio (MacRae and Lunn, 2006). Recent study on a series of TILLING mutants with smaller changes in AGPase activity demonstrated that AGPase exerts control over the pathway of starch synthesis (Hädrich et al., 2011). Moreover, the sugar trehalose-6-phosphate (Tre6P) has been proposed to act as an intermediate between sucrose and AGPase (Kolbe et al., 2005), which might provide the route whereby starch accumulation is linked to the sucrose and, possibly, plant carbon status (Smeekens et al., 2010; Stitt et al., 2010).

An important question concerning the capacity of a source leaf is whether the photosynthetic activity is always running at its maximum or is rather controlled by the metabolism of photoassimilates within, or their transport to, sink tissues. There are several examples of sink-dependent alteration of photosynthesis of source leaves. The overexpression of a sucrose phosphate synthase (*SPS*) gene in tomato caused considerable modification of carbon allocation within the leaves and additionally at the whole plant level. Most importantly, the amount of sucrose unloaded into the fruit was considerably higher, which lead to increase the total fruit number as well as total fruit weight (Micallef et al., 1995). Thus, suggesting that not only development, but also fruit growth are limited by sucrose available from phloem unloading. Consistently with this hypothesis, transgenic tomatoes with reduced sucrose synthase (SuSy) activity that catalyzes the cleavage of sucrose into UDP-glucose and fructose, displayed a reduced sucrose unloading capacity, leading to reduced fruit set as well as slower growth rate (D’Aoust et al., 1999). The negative effect of suppression of SuSy on fruit set is reminiscent of that on seed development observed in SuSy deficient maize (Chourey et al., 1998) and cotton (Ruan et al., 2008). Similarly, tomato plants constitutively overexpressing the *hexokinase 1* gene of *Arabidopsis* displayed reduced photosynthetic rates as well as harboring smaller fruit containing lower sugar content (Dai et al., 1999; Menu et al., 2004).

Tomato fruits initially contain chloroplasts that are photosynthetically active, but these differentiate to non-photosynthetic chromoplasts during ripening. This transition would appear to be coupled to a decline in the expression of genes (Alba et al., 2004; Carrari et al., 2006; Kahlau and Bock, 2008; Osorio et al., 2011) and enzyme activities (Schaffer and Petreikov, 1997; Steinhauser et al., 2010) associated with carbon assimilation. Despite the high expression of photosynthetic genes (Lemaire-Chamley et al., 2005), fruit are rarely net assimilators of CO_2_ (Carrara et al., 2001). Moreover, the triose phosphate and glucose-phosphate transporters are both active in tomato chloroplasts, indicating that both could import and export phosphoesters. Early studies shown that tomato fruit contributes by its own fixed carbon between 10 and 15% of the carbon skeletons required (Tanaka et al., 1974). A similar effect was also observed in transgenic tomato with reduced chloroplastic fructose-1,6-biphosphatase activity and thus likely reduced rates of fruit photosynthesis (Obiadalla-Ali et al., 2004). Moreover, the combined metabolomic and transcriptomic analyses of tomato plants with reduced expression of *Aux/IAA* transcription factor *IAA9*, suggested an important role for photosynthesis of ovary in the initiation of fruit development (Wang et al., 2009). A recent study showed that fruit-specific reduction in the expression of *glutamate 1-semialdehyde aminotransferase* (*GSA*), which has previously been documented to contribute to the control of chlorophyll biosynthesis (Höfgen et al., 1994), displayed lower chlorophyll levels and photosynthetic activity but few other differences. Indeed, no differences in fruit size, weight, or ripening capacity and only minor alterations in other primary or intermediary metabolites were observed (Lytovchenko et al., 2011). These results, suggest that fruit photosynthesis is not essential for fruit energy metabolism. However, the same study intriguingly demonstrated that fruit photosynthesis was important for seed set, indicating that further study is required to enhance our understanding of the interactions between different organs of the plant. Two recent studies provide highly intriguing insights into the competition for assimilates within the plant (Génard et al., 2010; Toubiana et al., 2012). The work of Toubiana et al. (2012), follows on from earlier work on tomato introgression lines which revealed clear negative correlations between fruit amino acid levels and the harvest index (the proportion of dry weight of the plant invested in, in this instance, its fruits; Schauer et al., 2006) even used pruning to simulate agronomic practice (Do et al., 2010). The latter study likely provides information with regard to environmental manipulations, which alter source-to-sink partitioning. That said much broader analysis such as those carried out in a collection of *Arabidopsis* accessions grown under a variety of C and N availabilities (Kleessen et al., 2012; Sulpice et al., 2013), will be required in order to achieve greater insight into the interplay between plant, environment, metabolism, and growth.

## TRANSPORT OF CARBOHYDRATES FROM SOURCE-TO-SINK

All photoassimilates that are not required for the support of leaf function are converted into sucrose or amino acids and loaded into the phloem for translocation to the sink organs. High concentration of sucrose in the sieve elements (SEs) of source tissues raise turgor pressure, resulting in hydrostatic pressure-driven mass flow of sugars to the SEs of sink tissues, where sugars are unloaded and turgor pressure drops. Sugar transport is highly regulated (Tiessen and Padilla-Chacon, 2013), and sucrose-specific signaling is involved in controlling transport activity (Chiou and Bush, 1998). Different transporters are required for efficient movement of sucrose across plasma membranes for apoplastic phloem loading in tomato source leaves and phloem unloading in fruit pericarp at the rapid expansion phase (Ruan and Patrick, 1995). These operate with different energetic and kinetic constraints rendering them suitable for: (i) efficient export into cell wall spaces, a process most likely mediated by sucrose facilitators such as AtSWEET11 and 12 (Chen et al., 2012), (ii) uptake of sucrose in cells as mediated by Suc/H^+^ symporters (Carpaneto et al., 2005), (iii) loading from the cytosol into storage vacuoles by hexose/Suc/H^+^ antiporters (Milner et al., 1995; Brown et al., 1997; Ruan et al., 1997), and (iv) fine-tuning of sucrose/hex flux in order to on the one hand maintain homoeostasis and on the other regulate intraorganellar signaling.

In tomato, three sucrose transporter genes have been identified, *LeSUT1*, *LeSUT2*, and *LeSUT4*. All three proteins were demonstrated to be co-localized in the SEs, whereas transcription of *SUT1* was also shown to take place in companion cells (Barker et al., 2000; Weise et al., 2000). *LeSUT1* is mainly expressed in sucrose exporting source leaves; whereas *LeSUT2* is expressed predominantly in sink organs such as sink leaves, stem, and fruits (Barker et al., 2000). Although the expression patterns of *LeSUT1* and *LeSUT2* are different at the tissue level, they are co-localized in the SEs in the loading and transport zone, particularly in leaves, petioles, and stem tissues. Moreover, both genes have been proven to physically interact, which is suggestive of the potential formation of oligomeric complexes with unique transport capacities (Reinders et al., 2002). However, oligomerization of the sucrose transporters has yet to be confirmed *in planta*, so the physiological importance of this observation is currently unknown. If sucrose transport mediated by these transporters is essential for phloem loading, then a reduction in transport activity would be anticipated to lead to feedback inhibition of photosynthesis and a consequent reduction in the supply of carbon to the sink organs. In order to test this hypothesis, transgenic tomato plants were generated which independently suppressed the expression of *LeSUT1* and *LeSUT2*. The leaves of *LeSUT1* antisense plants displayed early senescence and chlorosis. Furthermore, the rate of photosynthesis in these plants was reduced and analysis of metabolites revealed an accumulation of soluble sugars and the inability to mobilize transitory starch during prolonged dark treatment. Moreover, measurements of C eﬄux from cut petioles indicated a blockage in phloem loading a fact that rendered the plants unable to produce normal fruits (Hackel et al., 2006). By contrast reduced *LeSUT2* expression exclusively affected tomato fruit seed development, pollen germination, and pollen tube length. The data suggest, that *LeSUT1* and *LeSUT2* appear to have a role in phloem loading and unloading, respectively (Hackel et al., 2006).

The sucrose transporter of group 4 (*LeSUT4*) was originally published as high capacity transporter of phloem minor veins (Weise et al., 2000). However, the recent finding of other sucrose transporters from group 4 in *Arabidopsis* and barley tonoplasts (Endler et al., 2006), raises questions about the initial interpretation with it seeming more likely that group 4-type transporters are vacuolar sucrose transporters that are primarily expressed in sink tissues.

Recently, a new class of sugar transporters called SWEETs, which are involved in the release of sugars to the apoplast, have been described in *Arabidopsis* and rice (*AtSWEET 10–15*; *OsSWEET 11* and *14*; Chen et al., 2012). The *Arabidopsis* double mutant, *atsweet11;12*, displayed reduced export of carbon from the leaf, increased accumulation of starch, and a reduced photosynthetic capacity (Chen et al., 2012). The SWEET proteins have not yet been identified in tomato but can be predicted that they operate at the placenta–seed interface, in outer pericarp, at early developmental stages during hexose accumulation phase (Ruan and Patrick, 1995; Jin et al., 2009). Here, the Suc eﬄuxers likely facilitate the sucrose export into the apoplasm where Suc is hydrolysed by invertases into glucose and fructose prior to uptake by Hex/H^+^ symporters (Ruan et al., 2010). However, direct molecular genetic testing of this hypothesis is yet to be performed.

The importance of the supply to and the subsequent mobilization of sucrose in, heterotrophic organs has been the subject of considerable research effort spanning many years (Miller and Chourey, 1992; Zrenner et al., 1996; Heyer et al., 2004; Lytovchenko et al., 2007). While the mechanisms of sucrose loading into the phloem has been intensively studied over a similar time period (Riesmeier et al., 1993, 1994; Bürkle et al., 1998; Meyer et al., 2004; Sauer et al., 2004), those by which it is unloaded into the sink organ have only been clarified relatively recently (Bret-Harte and Silk, 1994; Viola et al., 2001; Kuhn et al., 2003; Carpaneto et al., 2005). In tomato, early studies suggested that sucrose unloading in pericarp during early stages of fruit development is likely symplasmic (Ruan and Patrick, 1995; Damon et al., 1998; D’Aoust et al., 1999). The post-phloem cellular pathway in the outer fruit pericarp was shown to shift from symplastic during starch accumulation (13–14 days after anthesis) to apoplastic during hexose accumulation (23–25 days after anthesis; Oﬄer and Horder, 1992; Ruan and Patrick, 1995; Patrick and Oﬄer, 1996). During the switch from the starch-accumulating to the sugar-accumulating phase, the symplastic continuity between phloem and storage parenchyma is diminished and an apoplastic unloading step for sucrose was thought to predominate. This symplastic-to-apoplastic switch is consistent with facilitated transport to accumulate soluble sugars at high concentrations without attenuating phloem unloading due to osmotic effect exerted from the recipient sink cells (Oﬄer and Horder, 1992). Three sugar transporter genes (acc. numbers: U321367, U336512, U318421) were found to co-localize with quantitative trait locus (QTLs) for sugar accumulation in tomato fruit (Prudent et al., 2011). Moreover, cultivar differences in hexose content of tomato fruit correlate well with maximal activities of hexose/H^+^ symporters (Ruan et al., 1997). This relationship was verified by RNAi knockdown of three hexose symporters (McCurdy et al., 2010) which localize to plasma membrane of storage parenchyma cells (Dibley et al., 2005). The reduction in fruit expression levels of these three hexose symporters caused a decrease in fruit hexose accumulation. By contrast, photoassimilate production by source leaves and phloem transport capacity to fruit were unaffected (McCurdy et al., 2010).

## CARBOHYDRATE METABOLISM AND ACCUMULATION IN SINK TISSUES

Experimental manipulations of source supply, source activity, and sink strength have all provided strong evidence for the hypothesis that photosynthesis and sink utilization of carbohydrates are tightly coordinated (Paul and Foyer, 2001; Kaschuk et al., 2010). Generally, when sink activity is decreased by removing active sinks or introducing nutrient deficiency, carbohydrates accumulate in leaves and photosynthesis becomes inhibited (Paul and Pellny, 2003), which does not depend on the sink removal, but on the remaining sink capacity. Similarly, when sucrose export from source leaves is restricted, photosynthesis is inhibited (Krapp and Stitt, 1995; Bürkle et al., 1998; Zhang and Turgeon, 2009), which is due to the remaining transport capacity. Therefore, the amount of sugars accumulated in fruit is not only dependent on endogenous metabolic processes but also in the degree of phloem unloading, since tomato fruits have a low photosynthetic activity (Farrar and Williams, 1991) which is actually not even required to support fruit growth (Lytovchenko et al., 2011).

As mentioned above some studies postulate apoplastic unloading from phloem throughout tomato fruit development (Zanor et al., 2009), especially during phases of hexose accumulation (Ruan and Patrick, 1995). In such a scenario, cell wall invertase catalyzes the breakdown of sucrose into glucose and fructose in the apoplasm, which have the potential to regulate sugar fluxes by increasing apoplasmic levels of hexoses. The apoplasmic unloading of sugar can thus facilitate influx of hexoses across plasma membrane of storage cells or can accelerate the eﬄux of sucrose from phloem to sink apoplasm by sucrose concentration differences, possibly mediated by SWEET sucrose eﬄuxers, as recently reported in Arabidospis and rice (Chen et al., 2012). The *Solanum pennellii* apoplasmic invertase (LIN5) identified as QTL for hexose accumulation in the tomato introgression line (Brix9-2-5), was characterized by a higher affinity for sucrose. Thus tomato containing the *S. pennellii* LIN5 exhibited a significant increase in soluble solids (usually sugars and acids) without a negative impact on fruit yield (Fridman et al., 2002, 2004; Gur and Zamir, 2004; Zanor et al., 2009). Increasing the activity, by silencing its inhibitor (*INVINH1*), similarly increased fruit sugar level and seed size (Jin et al., 2009). The metabolic rationale behind the strategy of apoplasmic invertase modification is that the hydrolysis of translocated sucrose at the point of unloading in the fruit sink can increase the gradient of translocation from source-to-sink and hence the net import into the fruit (Ho, 1996; Fridman et al., 2004; Koch, 2004). In addition, such a strategy has the added advantage that it generates glucose signals, which stimulate cell growth and sugar accumulation (Jin et al., 2009). By contrast to the effects from developmental regulation of cell wall invertase activity, constitutive knockdown of a vacuolar soluble acid invertase SAI (*TIV1*) caused a switch from hexose-accumulation to sucrose-storing tomato fruit without any change in total sugar content per fruit (Klann et al., 1996). Sucrose-accumulating fruits were smaller than control fruits, which suggest that soluble acid invertase controls sugar composition and cell expansion, consistent with the function of SAI in other plant systems (Roitsch and Gonzalez, 2004).

AGPase is a key regulatory plastidial enzyme of starch biosynthesis and maps to a QTL for sugar content (Petreikov et al., 2009). A tomato introgression line of *S. habrochaites* was characterized by increasing sugar content and higher AGPase activity that resulted from temporally extended expression of AGPase large subunit (Petreikov et al., 2006). These tomatoes exhibited higher starch content in the immature fruit, which leaded to higher total soluble solids (mainly sugar) and fruit size at mature stage (Petreikov et al., 2006). The relationship between AGPase activity and tomato sugar content has been verified altering malate metabolism, which post-translationally affects AGPase activity through an effect on cellular redox balance (Centeno et al., 2011; Osorio et al., 2013). Moreover, while modification in malate metabolism in tomato fruit had relatively little effect on the total fruit yield, they had dramatic consequences in postharvest shelf life and susceptibility to bacterial infection, which is an important consequence of altered C partitioning (Centeno et al., 2011).

Nowadays, there is an important debate whether or not C storage should be considered as an actively regulated sink instead of being a simple surplus resulted when supply of new assimilated carbon is higher than demands. To date, our understanding of the regulation of storage is based on diurnal starch dynamics, where starch is accumulated during the day to support growth and respiration at night. Therefore, from these studies can be concluded that the synthesis and degradation of starch are controlled by independent regulatory networks that allow plants to balance carbon supply via photosynthesis with C use for growth and other activities (Sulpice et al., 2009; Stitt and Zeeman, 2012). Although this question has not been opened in tomato plants, in long-lived trees recent attempts at explaining C limitation under stress suggest that priority allocation to storage could compete with growth and make assimilated C a limiting resource (McDowell, 2011; Sala et al., 2012). This considerations imply that under limiting availability of assimilates, C storage is given priority over growth, because ultimately survival depends more on C demands for metabolism than for growth (Sala et al., 2012). However, further empirical evidences are needed to corroborate these theories.

## SOURCE–SINK REGULATION BY STRESS

Plants are able to perceive and respond to a wide range of biotic and abiotic stimuli (Metlen et al., 2009). In response to these stimuli they undergo physiological, biochemical, and physical changes to produce a phenotype that match their environment (Sultan, 2000). Such phenotypic plasticity can be expressed locally at the site affected by stimuli. However, plants can also coordinate their responses to changes in their surroundings with other plant modules and respond in a systemic and integrated manner at the whole-plant level (de Kroon et al., 2005).

Upon attack by herbivores, plants produce a number of defensive compounds and structures that hinder the performance and fitness of the attackers. Several studies have shown that herbivore attack leads to reallocation of carbon and nitrogen from damaged leaves into storage tissues (Babst et al., 2005, 2008; Schwachtje et al., 2006), often in a rapid manner known as induced sequestration (Orians et al., 2011). In tomato specifically, export of nitrogen from leaves into roots has been shown in response to methyljasmonate (Gómez et al., 2010). Recently, the whole-plant metabolic responses of tomato after leaf herbivory by two caterpillars (the generalist *Helicoverpa zea* and the specialist *Manduca sexta*) were characterized using metabolic analysis (Steinbrenner et al., 2011). In this study, it was found that the primary metabolic responses across the entire tomato plant varied widely from tissue to tissue. The induced metabolic change was stronger in the apex and root tissues than in undamaged leaflets of damaged leaves, indicating rapid and significant whole-plant responses to damage. Interestingly, these metabolic changes were herbivore-specific, which *H. zea* herbivory strongly affected concentrations of defense-related metabolites, while *M. sexta* altered metabolites associated with carbon and nitrogen transport (Steinbrenner et al., 2011; Gómez et al., 2012).

Stresses including insufficient supply of nutrients, drought, heat, or cold, often induce seed and fruit abortion and, hence, irreversible yield losses (Boyer and McLaughlin, 2007; Bita et al., 2011; Li et al., 2012; Ruan et al., 2012). For example, heat stress can result in 70% yield loss in tomato as a result of flower and fruit abortion (Bita et al., 2011). If several enough, heat stress can also led to 100%, or to 10% if it is only mild (Sato et al., 2000). Therefore, any exogenous factors, which alter the resource availability from source and its utilization within sink, can be anticipated to influence carbohydrate partitioning, and sink yield and quality.

Heat mainly affects the biochemical reactions of photosynthesis, and depending on the duration and intensity, can irreversible damage Rubisco, oxygen-evolving complexes, chloroplast ultrastructure, thylakoid membranes, and PSII reaction centers (Havaux, 1993; Camejo et al., 2005, 2006). Tomato has an optimum growth temperature of 24–26^∘^C in the day and 18–20^∘^C at night. Temperatures above 30^∘^C in the daytime and 21^∘^C at night as well as lower that 15^∘^C could block the reproductive (gametophytic) phase in flowering plants, resulting in low pollen viability, poor pollen elongation, and ultimately fruit abortion (Weaver and Timm, 1989). A recent study has revealed that the reproductive development of tomato is more sensitive to high night temperature than day temperature (Liu et al., 2012). This implies that lack of photoassimilate supply at night aggravates heat-induced damage. A pollination of heat-stressed and emasculated flower with non-stressed pollen reduce the flower abortion rate, which indicate that pollen development is more vulnerable to heat stress than the female organs in tomato (Ruan et al., 2010). The reduction of tomato pollen viability by heat could be attributed to reduction in starch accumulation in developing pollen grains and total soluble sugar in the anther wall (Pressman et al., 2002). Further analysis by the same group revealed that reduction of cell wall invertase activity in anthers might be the major factor contributing to pollen sterility under heat stress (Pressman et al., 2006). This reduction in the cell wall invertase activity could be due to an induction of the invertase inhibitor protein (Frank et al., 2009). Interestingly, expression of SPS was also up-regulated by heat in maturing tomato pollen (Frank et al., 2009). SPS is a key enzyme in the biosynthesis of sucrose, which is thought to play an important role as osmo-protectant in the maintenance of cell membrane integrity and thereby cellular function. Recently, Li et al. (2012) described the influence of heat stress on fruit and seed set, a critical phase for realizing yield potential. They examined patterns of carbon allocation and sucrose cleavage enzymes in heat-tolerant and -sensitive tomato lines finding a strong correlation between high invertase activity and increased sucrose import into young fruit, and heat-tolerance most likely due to an increase in sink strength and sugar signaling activities (Li et al., 2012).

Drought induces large alterations in source–sink relations due to a modification of growth priorities and to a reduction of the performance of photosynthetic organs (Vu et al., 1999). For instance, water stress could inhibit fruit growth as a result of both sink and source limitations (Chaves et al., 2009; Muller et al., 2011). Similarly to the response of plants to heat and cold stresses, the reproductive phase in flowering plants is often highly sensitive to drought stress (Guilioni et al., 1997; Smith and Stitt, 2007). Some studies have addressed the regulation of source- and sink-specific enzymes in response to water deficit. In this vein, several studies demonstrated that the reduced expression of cell wall and vacuolar invertases in drought stress could promote abortion (Andersen et al., 2002; McLaughlin and Boyer, 2004; Zanor et al., 2009). However, it is important to note that these studies were merely correlative and that changes in hormone levels (Andersen et al., 2002) and in the expression of a diverse range of other genes (Boyer and McLaughlin, 2007) have also been reported to occur coincidently to abortion. Tomato transformants deficient in the expression of the cell wall invertase gene, *LIN5*, showed a higher incidence of abortion (Zanor et al., 2009). This suggest that the reduction in apoplasmic invertase activity is likely an early step in the signal transduction cascade linking perception of stress to the initiation of senescence and membrane degradation events that lead to irreversible abortion (Zanor et al., 2009). The changes documented in these transformants in the expression of genes associated with hormonal synthesis and function, provide hints to the nature of this cascade which may ultimately lead to its elucidation. It was recently shown that the reduced activity of another apoplastic enzyme, ascorbate oxidase, correlated with increased final fruit yield under drought stress (Garchery et al., 2013). This manipulation resulted in increases in stomatal conductance in leaf and sugar content, as well as a modified apoplastic hexose:sucrose ratio with the authors arguing that the increased redox state of the apoplast protects against the rise in reactive oxygen species (ROS) levels following stress. Therefore, ascorbate oxidase may be a good candidate for strategies aimed at improving water stress tolerance in tomato.

The detrimental effects of salts result not only from a water deficit due to the relatively high solute concentrations in the soil but also from specific Cl^-^ and Na^+^ stresses. The physiology of the tomato in salty and non-salty conditions has been extensively studied, revealing an inhibition in growth and development, respiration, and protein synthesis as well as disruption in nucleic acid metabolism and an increase in oxidative stress (Zhang and Blumwald, 2001; Jiménez et al., 2002; Gautier et al., 2009; Manaa et al., 2011, 2013). Additionally, in salt-treated plants, stomatal closure caused by depletion of cellular water content and the reduction in the transport of assimilates are the main causes of photosynthesis inhibition (Hare et al., 1998). Moreover, accumulation of glucose, fructose, and mainly sucrose in leaves as well as in ripe tomato fruits can also lead to a decrease in photosynthesis by feedback inhibition mechanisms (Poljakoff-Mayber and Lerner, 1994; Gautier et al., 2010). Furthermore, salinity leads to osmotic stress due to depletion of cellular water (Hare et al., 1998). This osmotic adjustment could lead to an accumulation in the vacuole of compatible solutes and ions, thus increasing the turgor potential (Romero-Aranda et al., 2001). However, an increase in the turgor pressure is not always related with an increase in water content in the cell, as cell size has also been documented to be reduced under conditions of salinity. Romero-Aranda et al. (2001) observed that it reduced cell expansion in tomato plants, where it was associated with a reduced osmotic and water potential and an increase in the turgor potential. Several cellular processes involved in salt–stress tolerance including osmotic adjustment, osmo-protection, ion homeostasis, elimination of oxygen scavengers, stress response are linked with the duration of the stress (Munns et al., 2002). Intriguingly, application of exogenous calcium has a mitigating effect on tomato fruit by salinity where it seems to induce adaptation via the activation of the enzymes involved in energy and carbohydrate metabolism (Gautier et al., 2009; Manaa et al., 2013). However, considerable further research is required in order to define the mechanisms by which calcium mediates this impact on metabolism and growth.

Plants grown for long periods at elevated [CO_2_] show a down regulation of leaf photosynthesis (Delucia et al., 1985; Sage et al., 1989), and carbohydrate source–sink balance is believed to have a major role in the regulation of photosynthesis through feedback inhibition (Stitt, 1991). Source–sink imbalance may occur during exposure to elevated [CO_2_] when photosynthesis rate exceeds the export capacity or the capacity of sinks to use photosynthates for growth, resulting in an accumulation of carbohydrates in photosynthetically active source leaves (Stitt, 1991). As we have previously mentioned, levels of soluble sugars in plant cells have been shown to influence the regulation of expression of several genes coding for key photosynthetic enzymes (Koch, 1996; Pego et al., 2000). The buildup in carbohydrates may signal the repression, but does not directly inhibit the expression, of Rubisco and other proteins that are required for photosynthesis (Stitt, 1991; Jang and Sheen, 1994; Makino and Mae, 1999). In tomato, transcript levels for Rubisco subunits, chlorophyll *a/b* binding protein (*Cab*), and Rubisco activase (*Rca*) decline with CO_2_ enrichment, whereas those for core proteins in phosystems I and II remain unchanged (Van Oosten et al., 1994; Van Oosten and Besford, 1995). Also, despite a large accumulation of starch occurring in leaves of elevated CO_2_ grown plants, transcript levels for AGPase show little change (Van Oosten et al., 1994). Furthermore, although photorespiration decreases under elevated [CO_2_] (Stitt, 1991) responses of enzymes and/or transcripts associated with the photorespiratory pathway have not been well investigated (Moore et al., 1999).

The response of plant growth to phosphorus (P) limitation is shaped differently from the response to nitrogen (N) limitation (Burns et al., 1997; De Groot et al., 2001). An explanation for this different response may be due to different function of N and P in the cell. N is part of the machinery of the plant’s energy metabolism (photosynthesis and respiration), whereas a relatively large part of inorganic phosphate is incorporated in structural compounds (phospholipids, nucleic acids; Mengel and Kirkby, 1987). N limitation affects CO_2_ fixation directly through effects on photosynthetic components rich in nitrogen such as chlorophyll, light-harvesting complex, and Rubisco (Hikosaka, 1996; Evans and Poorter, 2001). Furthermore, N limitation may affect CO_2_ fixation indirectly due to the limitation of growth and the subsequent accumulation of carbohydrates and feedback limitation of photosynthesis (Paul and Driscoll, 1997; De Groot et al., 2001). P limitation as well as N limitation, affects photosynthesis but though different mechanisms (De Groot et al., 2003; Fujita et al., 2003). This P limitation may affects photosynthesis through changes in the activity of Calvin-cycle enzymes, RuBP regeneration and/or Rubisco activity as long as P plays an important regulatory role in starch and sucrose biosynthesis, Rubisco activation and is also part of ATP and NADPH/NADP^+^. To test these hypotheses, an elegant experiment was designed using tomato grown plants at low N, high N, low P, and high P at two irradiances (De Groot et al., 2003). The results were consistent with the hypothesis of N-limited produces an reduction of photosynthesis, possible by feedback from the leaf carbohydrate pool, while under P-limited conditions the production of assimilates is limited (De Groot et al., 2003). This evidence was strengthened by analysis of tomato plants grown in liquid culture under P starvation (Fujita et al., 2003). However, direct molecular evidence and information about the regulatory networks under N and P limitation remain to be defined.

## CONCLUSION AND PERSPECTIVES

Growth and development in plants are integrated processes in which primary assimilation in source tissues is balanced by the metabolic needs of heterotrophic sinks. In this review, we briefly provide evidence that assimilate partitioning plays a central role in balancing photosynthetic activity in the leaves with photoassimilate utilization and storage in sink. The data presented clearly demonstrate that molecular tools can be applied to study whole plant physiology in the context of carbon partitioning and yield manipulation. However, to elucidate the mechanisms that regulate source–sink relations, complementary experimental approaches are required which also take environmental and eco(physio)logical factors into account. It will also be crucial to improve our understanding of plant sugar metabolism and unravel the underlying network of highly flexible regulatory mechanisms, which underpin it in order to gain insight into source–sink regulation. In a future world of elevated atmospheric carbon dioxide concentration and environmental deterioration, enhancing the capacity for sucrose export and carbon utilization is an important component of maximizing or even merely maintaining photosynthesis and yield. That said the concepts outlined here do not merely reflect the challenges presented in understanding the interplay between plant and environment, and metabolism and growth in a crop species such as tomato but have broader implications for understanding these trade-offs in any plant species.

## Conflict of Interest Statement

The authors declare that the research was conducted in the absence of any commercial or financial relationships that could be construed as a potential conflict of interest.
